# Experimental investigation of diffusion flames with different baffle-plate air-hole diameters

**DOI:** 10.1038/s41598-026-38141-2

**Published:** 2026-02-20

**Authors:** Ebrahim S. Mohammed, Hamada. M. Gad, Ibrahim A. Ibrahim, Saad E. Habik, Mohamed M. Elsakka

**Affiliations:** 1 Mechanical Power Engineering Department, Faculty of Engineering, Port Said University, Port Said, 42526 Egypt; 2 Faculty of Industry and Energy, East Port Said University of Technology, Salam Misr City, East Port Said, 45619 Egypt; 3 Faculty of Engineering, East Port Said National University, Salam Misr City, East Port Said, 45619 Egypt

**Keywords:** LPG, Baffle plate, Air hole diameter, Temperature distributions, Species concentrations, Diffusion flame characteristics, Combustion efficiency, Energy science and technology, Engineering, Environmental sciences

## Abstract

Diffusion flames are widely used in industrial combustion systems; however, the influence of baffle-plate air-hole diameter on flame characteristics and combustion performance remains insufficiently quantified through experimental studies. The present work experimentally investigates Liquefied Petroleum Gas (LPG) diffusion flames stabilized by multi-hole baffle plates with varying air-hole diameters. Five baffle-plate configurations with eight radially distributed air holes were tested at a constant thermal load of 32 kW over air–fuel ratios (AFR) of 15–30, while flame stability, temperature distributions, flame length, species concentrations, and combustion efficiency were systematically measured. The experimental facility consisted of an integrated setup linking air and fuel supply lines to the baffle plate and combustor chamber. The study involved the development of an empirical relation expressing flame length in terms of air-hole diameter (d_a_) and AFR, where the discrepancy between predicted and experimental results averaged approximately 2.5%. Combustion efficiency decreased with increasing air hole diameters. Specifically, the d_a_ increased from 10 mm to 15 mm, the combustion efficiency dropped by approximately 10.17% at AFR = 15 and 11.04% at AFR = 20.

## Introduction

Combustion plays a vital role in generating thermal energy, which is widely utilized across various sectors such as residential heating, marine engines, automotive propulsion, aerospace applications, including ramjets, and residential heating. The principal goals of contemporary combustion research are to improve flame characteristics, mitigate pollutant emissions, and maximize overall thermal efficiency^1–5^. Extensive investigations have been conducted to optimize gaseous-fuel combustion with the dual aim of enhancing thermal efficiency and minimizing pollutant emissions. Diffusion flame systems, in particular, have broadened the use of numerous gaseous fuels, including hydrogen and Liquefied Petroleum Gas (LPG), due to their ability to sustain reliable combustion over diverse operating regimes^6–10^. These advancements have motivated further research into flame stabilization techniques, such as modifying the geometry of flame stabilizers and air distribution mechanisms.

Despite generally exhibiting lower efficiency and producing greater pollutant emissions, diffusion flames are often favored over premixed flames because of their enhanced stability under a wide spectrum of operating conditions. Consequently, extensive research efforts have been focused on enhancing the combustion efficiency of diffusion flames and minimizing their environmental impact through various design and operational modifications^11–13^. The turbulence and flow recirculation generated by flame stabilizers play a crucial role in enhancing the mixing of fuel and oxidizer, thereby significantly affecting the combustion process in the primary reaction zone downstream of the burner^14–16^.

Flame stabilization methods in combustion are generally categorized into four main techniques: the counterflow method^17,18^, pilot flame technique^19,20^, bluff body stabilization^21,22^, and air swirl stabilization^23,24^. Each technique employs unique mechanisms to sustain a stable flame front across a range of combustion conditions. Bluff body stabilization employs various geometric shapes to create effective flame anchoring zones. Common bluff body configurations include circular, triangular, Y-shaped, horizontal and vertical cylindrical, elliptical, conical, wall-blade, and V-gutter shapes^25–29^. These shapes generate wake regions and recirculation zones downstream, which enhance flame stability by trapping hot combustion products and promoting efficient mixing of reactants. In addition, baffle plates function as flow-disrupting elements that generate separated flow and recirculation zones, which are beneficial for flame stabilization. Unlike air swirlers, typically requiring intricate geometries and complex manufacturing, the baffle plate provides a more straightforward and cost-effective alternative. Its simplicity enables a more compact combustor configuration, making it particularly advantageous for space-constrained applications such as portable combustion devices and micro-scale power generation systems^30,31^.

Mola et al.^32^ conducted an experimental study examining how variations in nozzle diameter influence flame quenching behavior. They found that reducing the nozzle diameter narrows the operational flammability limits due to the increased mixture exit velocity. This elevated velocity intensifies flame stretch and turbulent mixing, thereby making ignition and stable flame propagation more difficult. Kim et al.^33^ numerically studied the effect of baffle shape and air hole size on flow structure in a combustor. Their results indicate that central flow recirculation plays a key role, and baffles with smaller air holes enhance mixing. From a thermal perspective, the study reports that smaller air hole diameters lead to an increase in flame temperature. Namazian^34^ used methane-air non-premixed flames to investigate the effect of air velocity achieved by decreasing air hole diameters on flame temperature. The results showed that higher air velocity raises the flame temperature when the fuel flow rate is kept constant.

Kim and Park^35^ conducted numerical simulations to evaluate stability limits under lean conditions, examining lean-limit extinction across various configurations. These included center–wall-blade, center-blade, wall-blade and multi-hole baffle designs. In addition, the center blade achieved the highest reduction (down to 37%). They further analyzed how variations in baffle-plate air-hole number (Na) influence performance and combustion characteristics. Increasing N_a_ from 3 to 10 led to an 8–20% improvement in combustion efficiency compared to a configuration with infinite holes. Kim and Park^36^ investigated a combustor equipped with multi-hole baffle plates to analyze the effect of varying the number of air holes (N_a_). This aims to investigate the combustion characteristics and heat transfer. The results showed that increasing N_a_ improved combustion efficiency by up to 20%, with favorable flame temperature distribution. Furthermore, shorter flame length had been achieved in the range of N_a_ between 5 and 8 holes.

Kim and Park^37^ numerically examined the air-hole geometry effect by comparing circular, square, and triangular configurations. Their results revealed that triangular openings yielded the shortest flame length, approximately 60% of that produced by circular holes. In a subsequent numerical investigation, they analyzed how rotating noncircular air holes influenced flame characteristics. The results showed that the wall temperature was most evenly distributed when the triangular openings were oriented at 15° and the square openings at 30°. In contrast, the minimum flame lengths were observed at 60° for square openings and 45° for triangular ones^38^. Additionally, Kim and Park^39^ had investigated the positioning of air-hole and its effect on performance of the baffle plate, finding that as the distance between the air holes and the fuel nozzle is increased, the development of a central recirculation zone is enhanced, whereas reducing this distance promoted wall-side recirculation dominance.

Moghtaderi et al.^40^ conducted tests on a micro-reactor fitted with a perforated baffle plate, evaluating its performance under non-reacting cold-flow conditions. The study highlighted the importance of air-hole placement, showing that positioning the radial holes at a distance of D/4 from the reactor center produced the most favorable flow characteristics. Similarly, Yahagi et al.^41^ performed an experimental investigation to examine the flame configurations that develop downstream of a baffle plate. The study revealed three distinct flame shapes corresponding to different Reynolds numbers of the premixed gaseous flow. At a Reynolds number of 400, the flame displayed a conical structure, which transitioned to a spherical shape as the Reynolds number increased to 800. When the Reynolds number reached 1200, the flame adopted a characteristic V-shape. These observations underscore the critical role of flow dynamics in determining combustion behavior and highlight the strong influence of flow conditions on flame morphology in confined combustion systems.

Although several numerical studies have examined the influence of baffle-plate air-hole diameter on flow structure and flame behavior, experimental investigations remain limited and are often restricted to cold-flow analysis or qualitative flame visualization. In particular, there is a lack of experimentally resolved data linking air-hole diameter to flame temperature fields, exhaust emissions (CO and NO), visible flame length, and combustion efficiency under controlled thermal load. This gap restricts the validation of numerical models and the practical optimization of baffled combustors. The present study addresses this gap by providing a comprehensive experimental dataset that quantifies the coupled effects of air-hole diameter and air–fuel ratio on flame dynamics, emissions, and thermal performance. Such experimentally resolved datasets are essential for validating numerical predictions and assessing the practical implications of air-hole diameter variations in real combustion systems. The resulting dataset is intended to serve as a robust benchmark for validating and refining future computational models. The experimental setup employs baffle plates with eight radially distributed air holes (N_a_ = 8), uniformly arranged at a radial distance of 50 mm. The study investigated air-hole diameters of 8, 10, 12, 14, and 15 mm. LPG was used as the fuel, and the air–fuel ratios (AFR) were set to 15, 20, 25, and 30. The thermal input was maintained at 32 kW, calculated from the product of the fuel mass-flow rate and its lower heating value.

The primary objectives of this work are to experimentally investigate the influence of baffle-plate air-hole diameter on the diffusion flame characteristics of LPG, including:

- Flammability limits.
- Temperature distributions.
- Maximum flame temperature.
- Axial flame temperature.
- Visible flame length.
- Species concentrations (O₂, CO₂, CO, and NO).
- Combustion efficiency.

## Experimental test rig

**Fig. 1:**
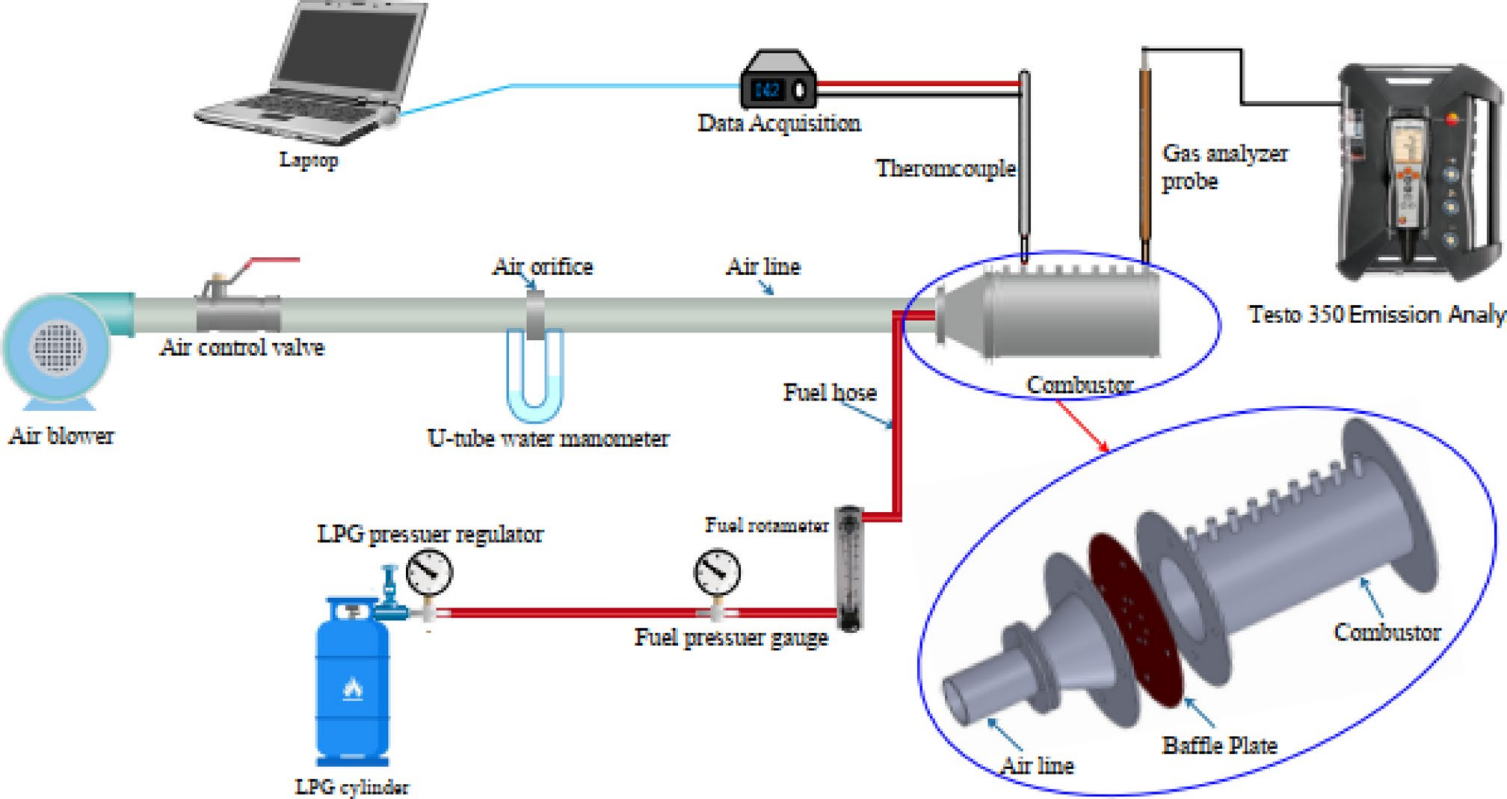
A schematic layout illustrating the configuration of the experimental test rig.

**Fig. 2:**
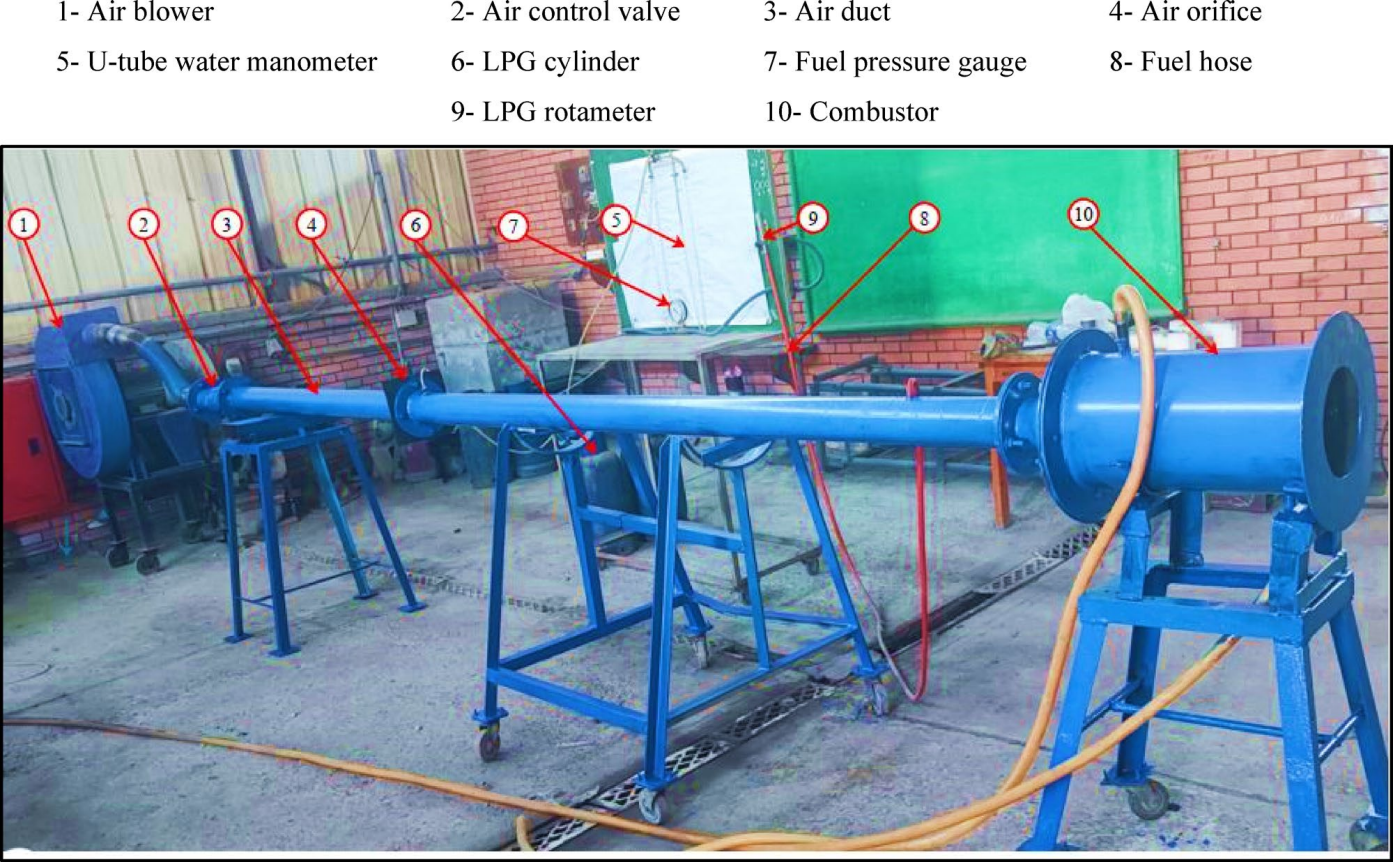
A photograph of the experimental test rig.

A dedicated experimental rig was developed to examine how variations in the baffle-plate air-hole diameter influence the behavior and performance of LPG-fueled diffusion flames. Figure 1 presents a schematic layout of the experimental setup, illustrating the air and fuel supply systems and their integration with the cylindrical combustor, while Fig. 2 provides a photograph of the assembled test rig used in the experiments. The system comprises two primary supply lines, air and fuel, both connected to the combustor. The air line includes a blower, a 100-mm-diameter duct, an orifice-type flow meter, a regulating valve, and a U-tube manometer for pressure measurement. The fuel system consists of an LPG cylinder equipped with a pressure regulator and a flexible supply line feeding the centrally mounted injector, with a rotameter and pressure gauge used to monitor the fuel flow rate. A constant thermal input of 32 kW was selected to ensure stable flame operation and repeatable measurements over the investigated range of air–fuel ratios and baffle-plate configurations, consistent with operating conditions reported in the literature^52^. In this study, the LPG blend used has a lower heating value of about 46.6 MJ/kg and a higher heating value of roughly 50.16 MJ/kg, and it consists of approximately 70% butane and 30% propane. Additional characteristics of the fuel include a vapor density of roughly 1.85 relative to air, an autoignition temperature spanning approximately from 410 to 580 °C, and a boiling temperature in the range from − 27 to − 20 °C^28,42– 43^.

**Fig. 3:**
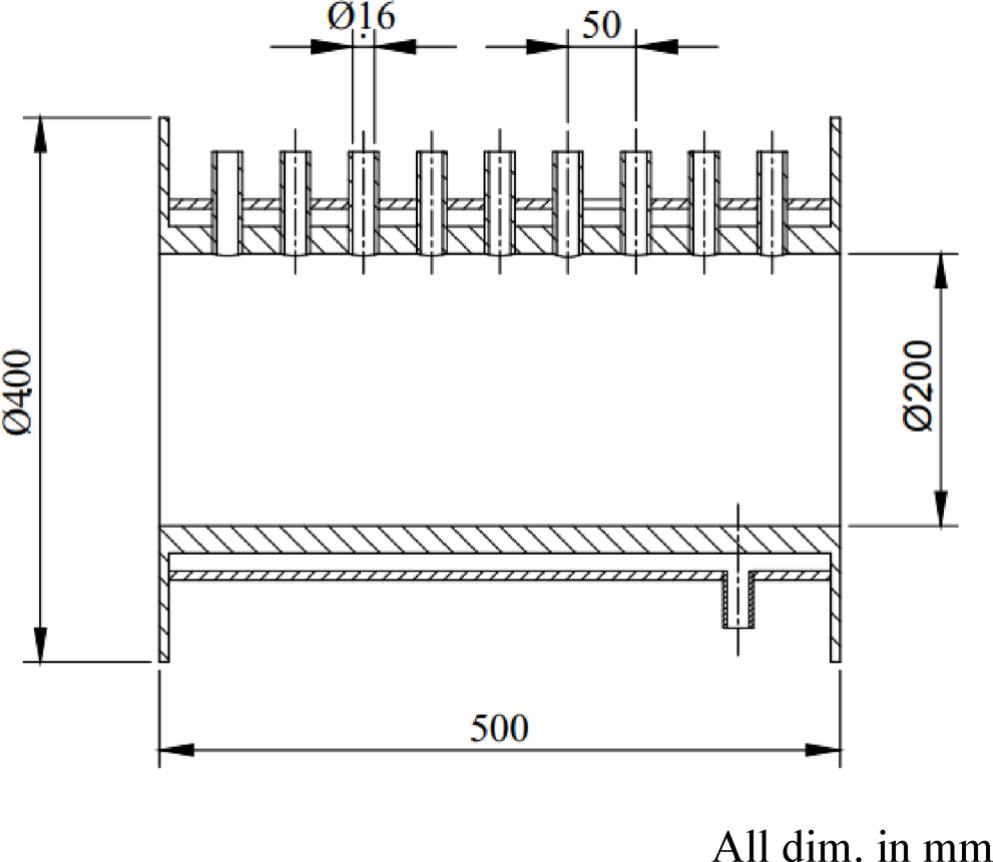
Geometric configuration and dimensional specifications of the combustor.

**Fig. 4:**
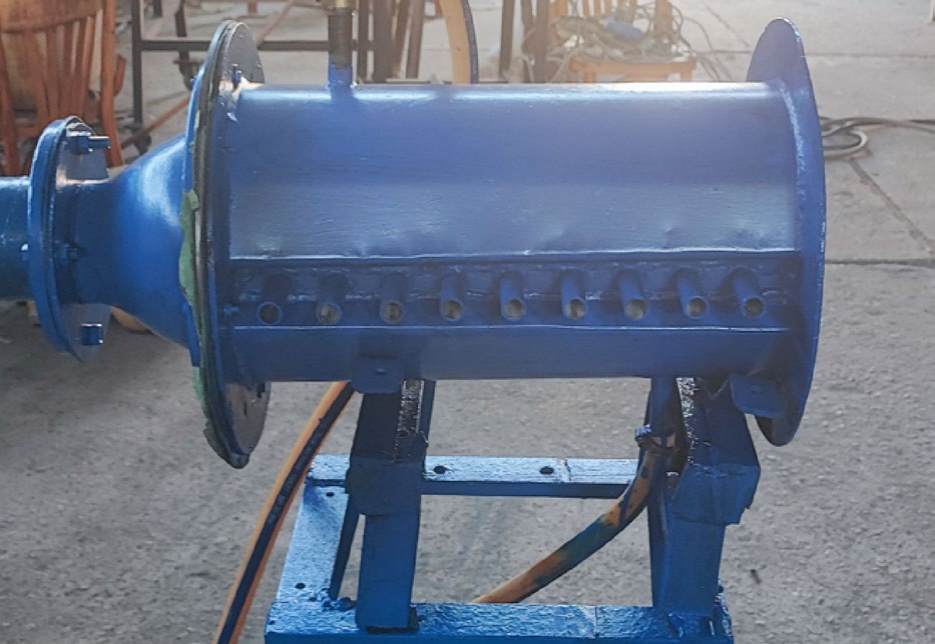
A photograph of the combustor.

The combustor consists of a steel cylindrical chamber with an internal diameter of 200 mm and a length of 500 mm and is equipped with nine access ports to facilitate measurements of visible flame length, temperature distributions, and exhaust species concentrations. Fuel is supplied through a centrally mounted injector with an internal diameter of 6 mm. Figure 3 presents the geometric configuration and key dimensions of the combustor, while Fig. 4 shows a photograph of the assembled chamber used in the experiments. The combustor system was designed as a general-purpose experimental rig to investigate fundamental flame characteristics rather than to replicate a specific industrial or domestic burner.

**Fig. 5:**
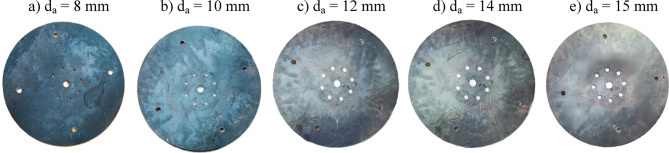
A photograph of baffle plates with different d_a_ at N_a_ = 8 and R_a_ = 50 mm.

**Fig. 6:**
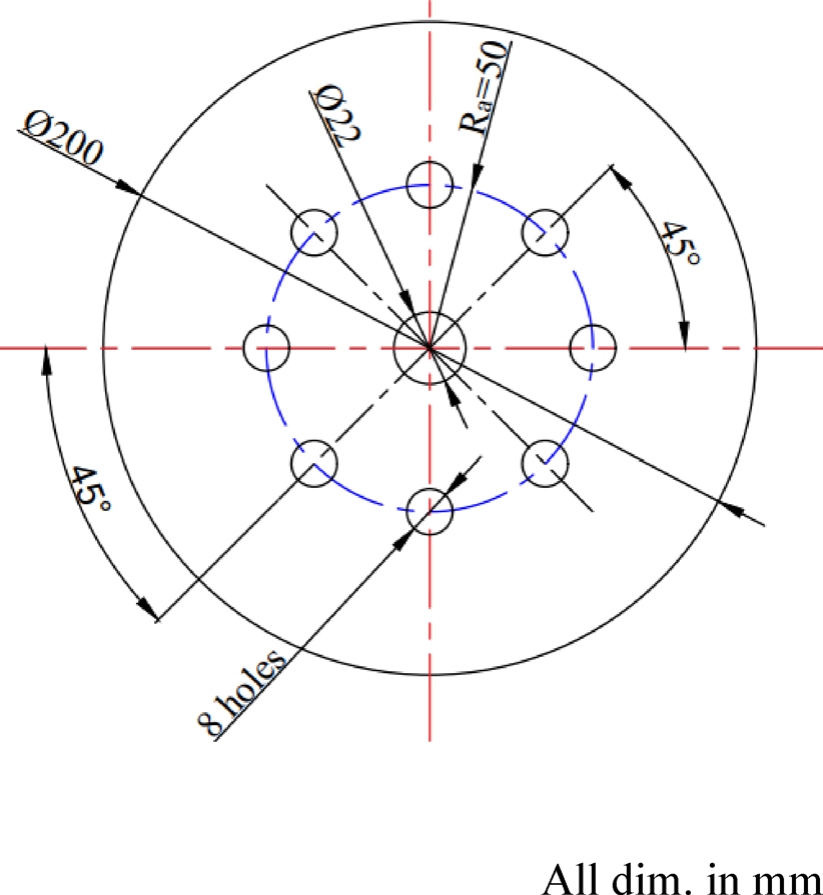
A schematic layout illustrating the configuration and dimensions of the baffle plate.

The baffle-plate configurations investigated in this study are shown in Fig. 5, which illustrates photographs of baffle plates with different air-hole diameters (dₐ) for a fixed number of holes (N_a_ = 8) and a radial hole position of R_a_ = 50 mm. The baffle plate incorporates a ring-shaped arrangement of air passages surrounding the centrally mounted fuel nozzle and plays a critical role in controlling flow recirculation, mixing, and flame stabilization. Five baffle plates, each with a thickness of 5 mm, were tested, featuring air-hole diameters of 8, 10, 12, 14, and 15 mm. For all configurations, eight air holes (N_a_ = 8) were uniformly distributed at a radial distance of 50 mm from the center. The detailed configuration and dimensional specifications of the baffle plate are provided in Fig. 6.

Air velocity in this study was measured using a calibrated orifice plate, with calibration performed earlier using a rotating vane anemometer (Testo 435; measurement range 0–40 m/s, accuracy ± 0.6 m/s). Flame-temperature distributions were recorded using a Type-R thermocouple with water cooling (Pt/Pt-13%Rh), capable of reliable measurements from − 50 °C to 1760 °C. A dedicated fuel rotameter was used to independently monitor the LPG volumetric flow rate. To limit radiation-induced errors, a fine thermocouple bead (~ 0.3 mm) was used to ensure accurate temperature measurements. Previous studies by Bradley and Matthews^44^, Shehata et al.^45^, and Pitts et al.^46^ reported that radiative and conductive heat losses are negligible under comparable combustion environments.

The uncertainty analysis ensures experimental reliability by accounting for potential deviations in measured quantities. It is generally known that the actual value of a measured quantity can never be obtained exactly, even when highly sensitive instruments are used. Therefore, in a single measurement, uncertainty can be determined using the standard deviation. The difference between the actual value and the measured one is known as the error, which can be classified as either systematic or random. This section aims to determine the possible maximum errors expected in the readings and, consequently, in the measured quantities.

Let the parameter R_s_ represent the result computed from the n measured, x_1_, x_2_,…, x_n_, the overall uncertainty interval is defined using the root-sum-square method,

1 $$\:\partial\:{\mathrm{R}}_{\mathrm{s}}={\left\{{\sum\:}_{\mathrm{i}=1}^{\mathrm{n}}{\left[\frac{\partial\:{\mathrm{R}}_{\mathrm{s}}}{\partial\:{\mathrm{x}}_{\mathrm{i}}}.\partial\:{\mathrm{x}}_{\mathrm{i}}\right]}^{2}\right\}}^{1/2}$$

and

2 $$\:\partial\:{\mathrm{R}}_{\mathrm{s}}\mathrm{\%\:=}\pm\:\frac{\partial\:{\mathrm{R}}_{\mathrm{s}}}{{\mathrm{R}}_{\mathrm{s}}}\mathrm{*}100$$

By applying Eqs. (1) and (2), the error in the measured quantities. Type-K thermocouples, operating between − 25 and 125 °C with a precision of ± 0.1 °C, were used to monitor the outlet and inlet temperatures of the combustor jackets cooling water, as well as the temperature of the combustor’s external surface. Under the flow conditions encountered in this work, the uncertainty associated with radiation correction ranged from 1% to 4%, which is sufficiently low to ensure a reliable temperature measurement. A Testo 350 gas analyzer was employed to measure species concentrations, with detection ranges of 0–50 vol% for CO₂, 0–500,000 ppm for CO, 0–20,000 ppm for NO, and 0–25 vol% for O₂. To ensure statistical validity, all experimental measurements were repeated under identical operating conditions, and the reported values represent time-averaged results after signal stabilization. Measurement uncertainty was quantified using the Mean Absolute Percentage Error (MAPE) method by accounting for the uncertainties of all contributing instruments. The resulting overall uncertainties were ± 2.5% for visible flame length, ± 3.5% for fuel flow rate, and ± 4% for air flow rate, which are sufficiently low to confirm the reliability and repeatability of the experimental trends reported in this study.

## Experimental results and discussions

In this study, each baffle-plate design includes eight air passages (N_a_ = 8) arranged radially at a distance of 50 mm (R_a_) from the center. The air-hole diameters (d_a_) examined were 8, 10, 12, 14, and 15 mm. Tests were performed to analyze the behavior of LPG diffusion flames at air–fuel ratios (AFRs) of 15, 20, 25, and 30, which were obtained by varying the air mass flow while keeping the LPG mass flow fixed at 0.71 g/s. Based on the using the aforementioned operating conditions., a constant thermal input is adjusted to be 32 kW. The analysis focuses on key combustion parameters, including ignition and extinction limits, temperature-field profiles, maximum flame temperature, overall efficiency performance, measured visible flame length, and major species concentrations. The subsequent subsections provide a detailed discussion of these parameters, which are summarized in Table 1 along with their corresponding ranges and operating conditions, highlighting their influence on flammability limits and flame characteristics.

**Table 1:** A summary of the studied parameters on flammability limits and the flame characteristics.

T. L., kW	AFR	*R*_a,_ mm	d_a_, mm	*N*_a_
-	Flammability Limits	50	8, 10, 12, 14, 15, 16 and 17	8
32	15 and 20		10	
	15, 20, 25, and 30		12	
			14	
			15	

### The flammability limits

**Fig. 7:**
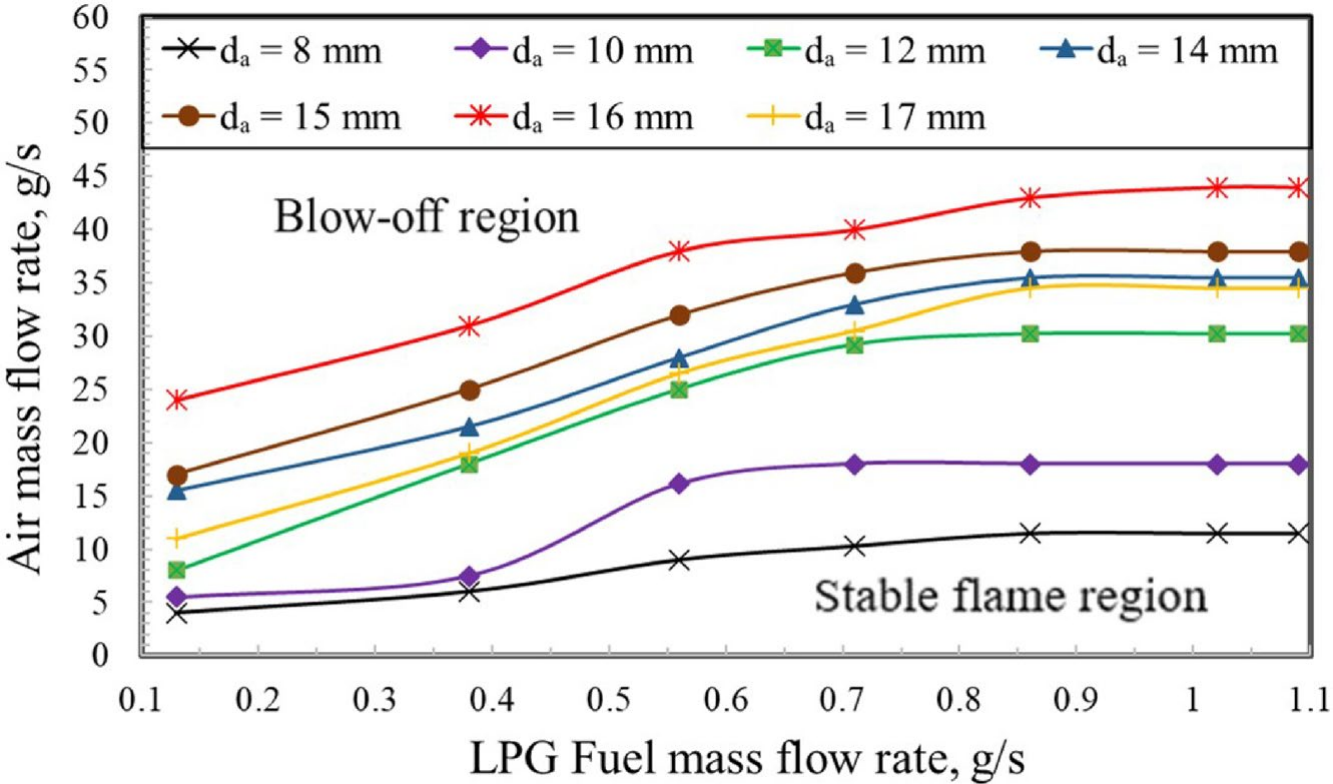
Variation of flammability limits with air-hole diameter for LPG-fuel.

In combustion systems, flammability limits represent a key parameter, defining the range of operating conditions under which a flame can be sustained without extinction and, therefore, identifying the window for stable combustion. As shown in Fig. 7, the stability and blow-off characteristics of the LPG diffusion flame are strongly dependent on the baffle-plate air-hole diameter. At a fixed fuel mass flow rate (ṁ_f_), flame blow-off occurs when the air mass flow rate exceeds a critical threshold, whereas stable combustion is maintained below this limit. The results indicate that increasing the baffle-plate air-hole diameter leads to a wider stable operating region, thereby enhancing flame stability over a broader range of air flow rates. This behavior is attributed to the reduction in air-jet exit velocity and flame stretch associated with larger air holes, which promotes improved flame anchoring and delays the onset of blow-off.

In the present experiments, the total air mass flow rate was maintained constant for each operating condition. Consequently, reducing the air-hole diameter leads to an increase in jet exit velocity and a corresponding increase in the Reynolds number. Despite this increase, the observed flame stabilization characteristics do not scale monotonically with Reynolds number. Instead, flame anchoring, stretch, and length are governed primarily by the jet momentum flux distribution and the resulting recirculation-zone topology downstream of the baffle plate. Smaller air-hole diameters produce higher-momentum jets and stronger shear layers, enhancing entrainment and promoting a dominant central recirculation zone that improves flame anchoring and increases flame stretch.

Specifically, within the LPG fuel mass flow rate range of approximately 0.12 to 0.45 g/s, it has been found that the stable flame zone becomes narrower, whereas it widens when the fuel mass flow rate increases from 0.45 to 1.1 g/s. The baffle plate with a 15 mm air hole diameter exhibits wider flammability limits compared to that with an 8 mm diameter. According to Mola et al.^32^, reducing the air hole diameter narrows the operational flammability limits due to the increased exit air velocity. This enhanced velocity intensifies flame stretch and turbulent mixing, making ignition and stable flame propagation more challenging. Therefore, an air hole diameter of 8 mm failed to achieve the required air–fuel ratios due to its narrowest flammability limit range. Meanwhile, the 10 mm air hole diameter exhibited a relatively limited flammability range, achieving air–fuel ratios of only 15 and 20. At an air hole diameter of 17 mm, a noticeable reduction in the flammability limits is observed. This reduction is attributed to the significant decrease in air exit velocity caused by the larger hole area, which lowers the mixing intensity and flame turbulence, thereby adversely affecting flame stability.

### The temperature distributions

A thorough characterization of the LPG diffusion flame necessitates measuring temperature distributions along both the axial and radial directions of the combustor. These measurements are presented as two-dimensional temperature maps, providing a clear visualization of thermal variations throughout the chamber. The analysis considers these temperature profiles for several baffle-plate configurations, namely, different air-hole diameters, across a range of test conditions that include multiple air–fuel ratios.

Flame temperatures were measured using a Type-R thermocouple, inserted radially through dedicated access ports and moved in 10-mm increments along the radius at several predefined axial locations in the combustor. The measured data were then processed using Microsoft Excel to create temperature contour maps, divided into twelve zones, each assigned a unique color representing a specific temperature range. This approach provides a clear visualization of the spatial temperature distribution and confirms the experimental nature of the measurements.

**Fig. 8:**
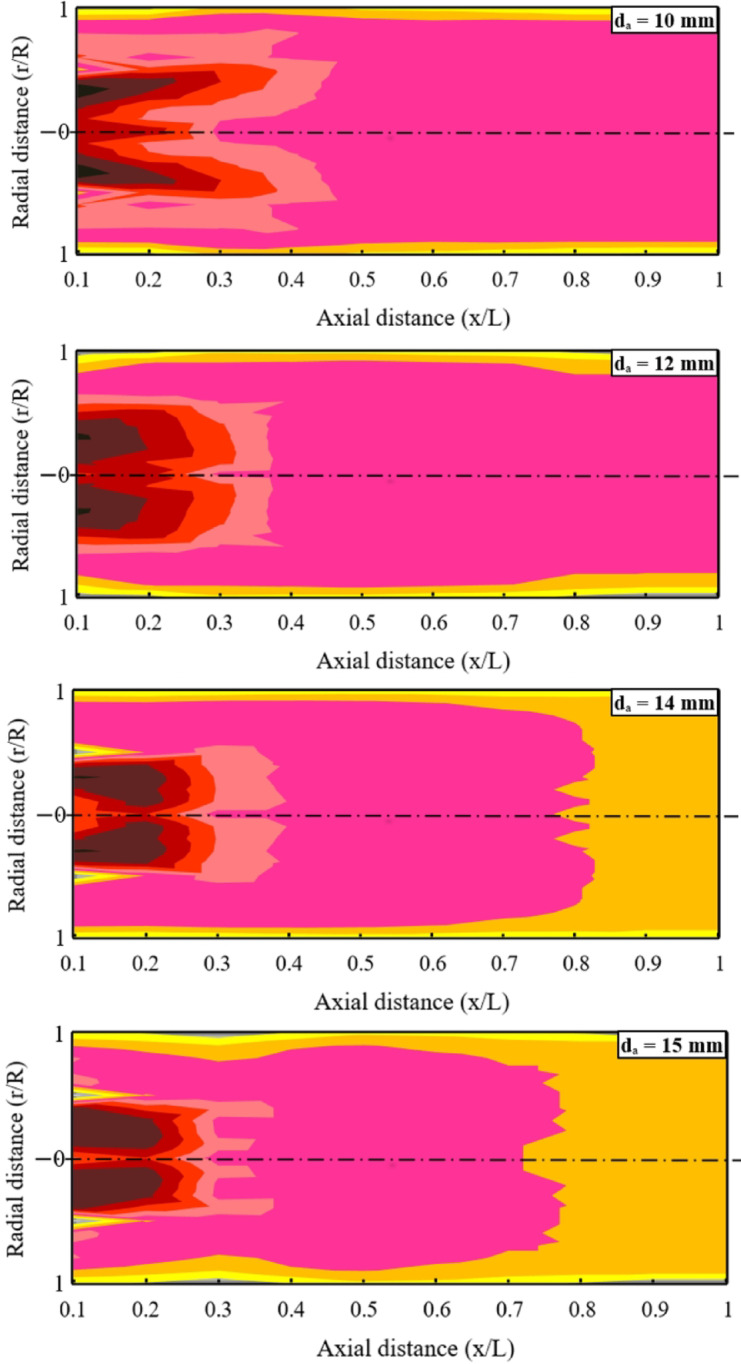
Effect of d_a_ on temperatures maps [AFR = 15 and T.L. = 32 kW].

As shown in Fig. 8, under an air–fuel ratio of 15 and a fixed thermal input of 32 kW, variations in the baffle-plate air-hole diameter have a pronounced effect on the flame-temperature field. Increasing the air-hole diameter from 10 mm to 15 mm causes the region of maximum temperature to shift upstream toward the baffle plate, indicating changes in mixing intensity and heat-release distribution near the plate surface. Previous studies have demonstrated that the air-hole diameter strongly governs the internal flow structure within the combustor^33,34^. Larger air holes promote wall-dominated recirculation along the inner combustor surface, which gradually becomes the prevailing flow pattern, whereas smaller air holes generate higher-velocity jets that strengthen the central recirculation zone and enhance fuel–air mixing. As the air-hole diameter increases, the associated reduction in jet momentum weakens the central recirculation region and shifts the flow structure toward wall-dominated recirculation, leading to increased dilution near the flame root and a reduction in local reaction intensity^35,39^. Consequently, from a thermal perspective, larger air-hole diameters result in a measurable decrease in flame temperature toward the combustor exit, primarily due to enhanced dilution and increased heat losses associated with stronger wall recirculation. The upstream shift of the high-temperature region indicates a redistribution of the main heat-release zone toward the baffle plate, resulting from enhanced wall recirculation and increased dilution of the reacting mixture downstream.

### Maximum flame temperatures

**Fig. 9:**
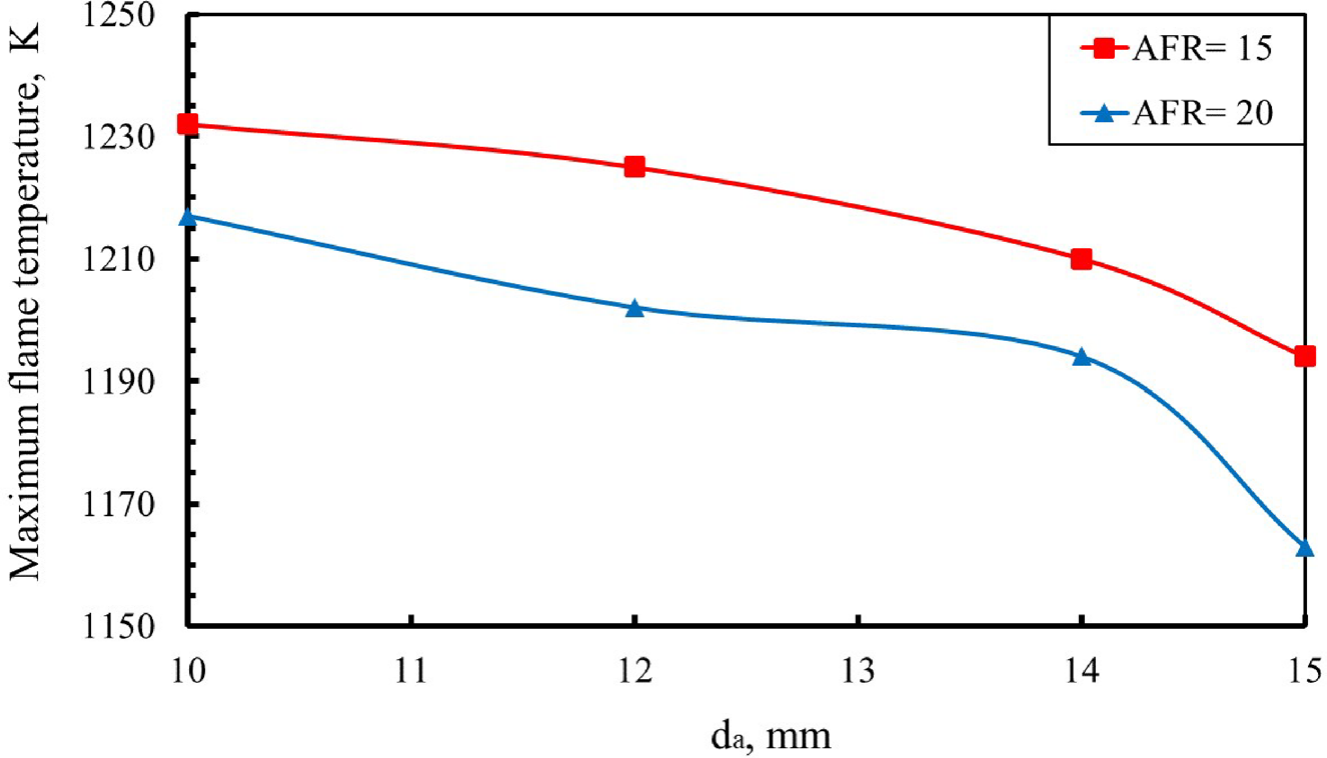
Influence of air-hole diameter d_a_ on maximum flame temperature across various AFRs.

In combustion science, accurate determination of the maximum flame temperature is essential, as it directly influences combustor material selection, overall combustion efficiency, and the formation of temperature-dependent pollutants such as NO_x_. As shown in Fig. 9, the peak flame temperature exhibits a clear dependence on the baffle-plate air-hole diameter. At an air–fuel ratio of 15, increasing the air-hole diameter results in a systematic reduction in the maximum flame temperature. This trend is consistent with the findings of Namazian^34^, who reported that increasing air velocity, corresponding to smaller air-hole diameters, leads to higher maximum flame temperatures at a constant fuel mass flow rate. Quantitatively, for the d_a_ = 10 mm at AFR = 15, the maximum flame temperature is approximately 0.6%, 1.8%, and 3.0% higher than those measured for d_a_ = 12, 14, and 15 mm, respectively. A similar trend is observed at AFR = 20, where the maximum flame temperature for d_a_ = 10 mm exceeds the corresponding values for d_a_ = 12, 14, and 15 mm by about 1.2%, 2.0%, and 4.5%, respectively.

### Axial flame temperatures

**Fig. 10:**
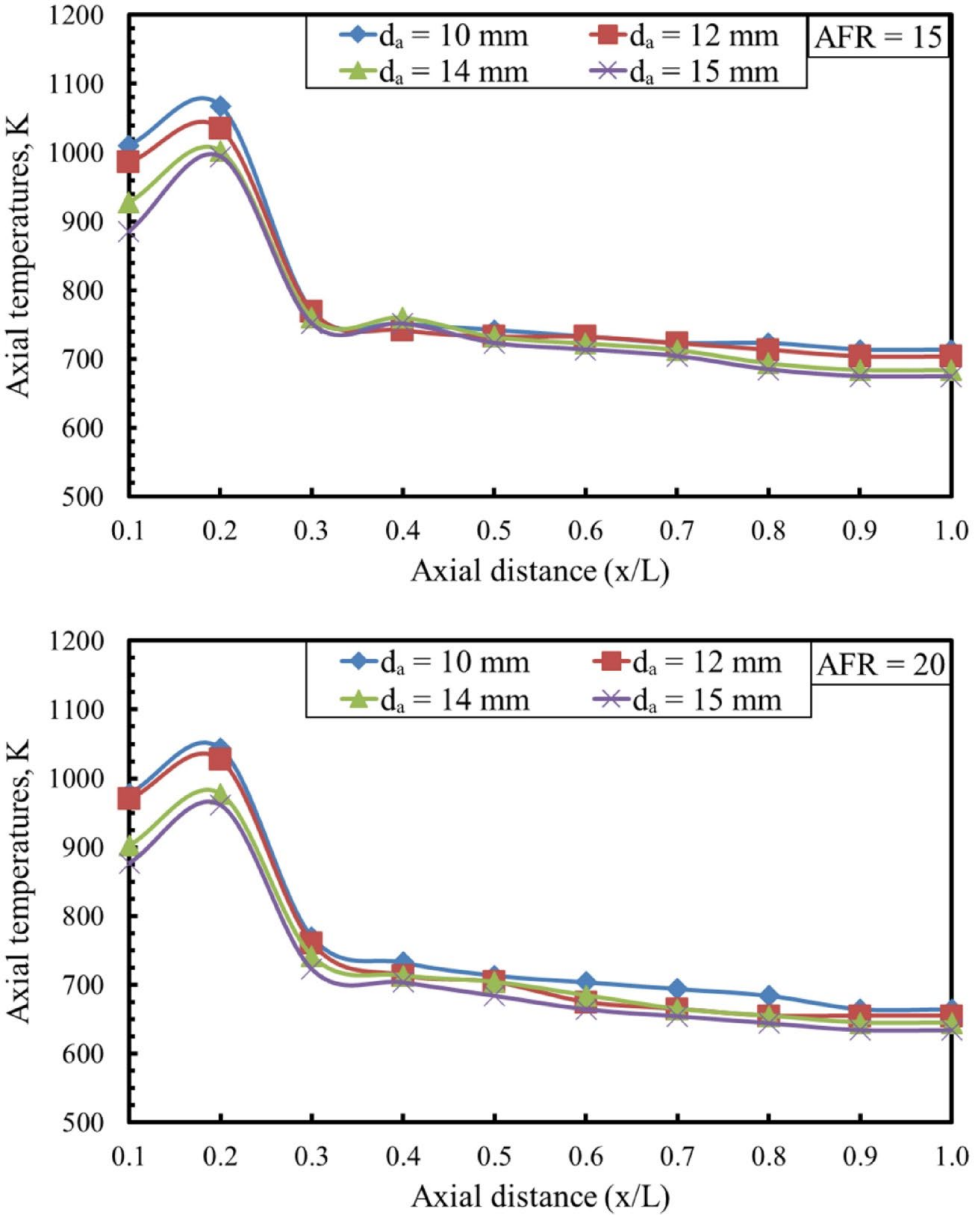
Effect of d_a_ on centerline axial flame temperatures for different AFR.

As shown in Fig. 10, variations in the baffle-plate air-hole diameter significantly influence the centerline axial flame temperature at air–fuel ratios of 15 and 20. For all configurations, the axial temperature along the combustor centerline increases with the dimensionless axial distance (x/L), reaches a maximum value, and then gradually decreases toward the combustor exit, indicating the completion of the combustion process. In both cases, an inverse relationship is observed between the air-hole diameter and the centerline axial flame temperature, with smaller air holes producing higher peak temperatures along the flame axis. This behavior can be attributed to the higher air-jet velocities associated with smaller hole diameters, which enhance mixing intensity, combustion rate, and thermal gradients, in agreement with the findings reported by Namazian^34^. Smaller air-hole diameters increase jet velocity and turbulent mixing rates, thereby accelerating chemical reaction rates and increasing the local heat-release intensity, which explains the higher peak flame temperatures observed. The subsequent decline in axial temperature beyond the peak location reflects progressive fuel depletion and dilution by excess air, marking the completion of the main combustion reactions along the flame axis.

### Visible flame length

**Fig. 11:**
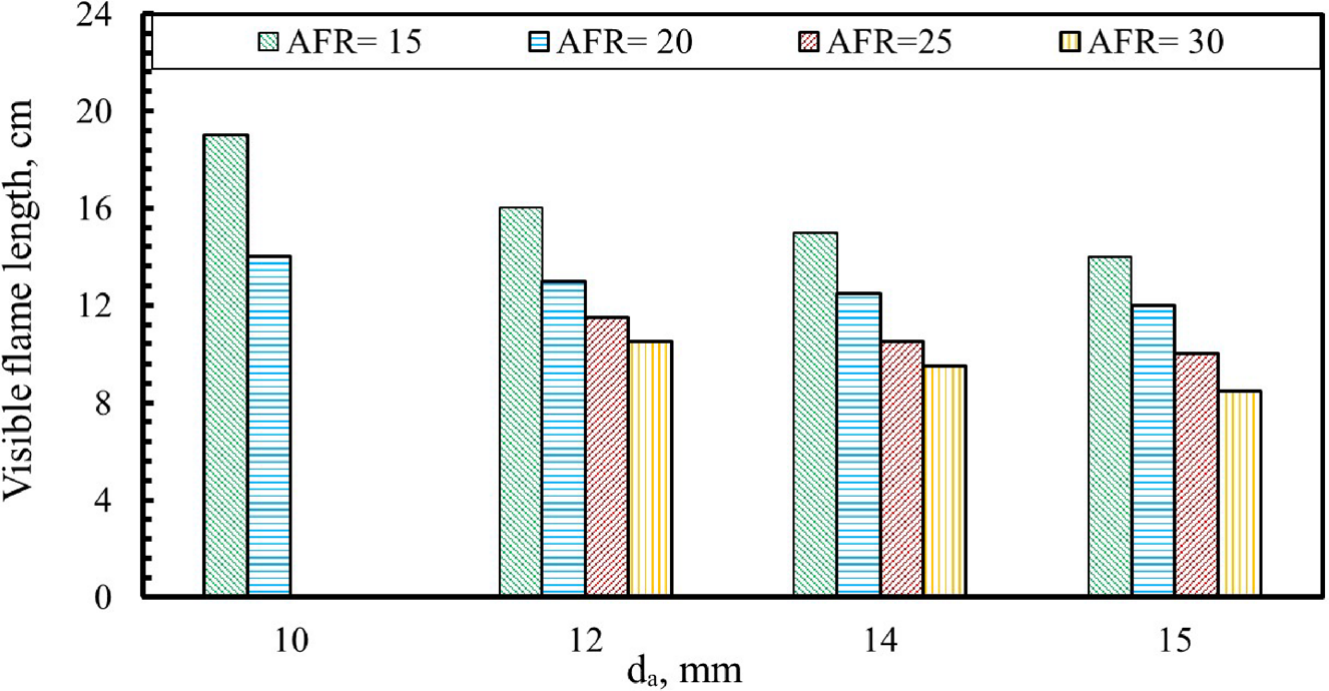
Influence of air hole diameter (d_a_) on visible flame length at different Air to Fuel Ratios (AFR) values [Thermal Load = 32 kW].

As shown in Fig. 11, the visible flame length is strongly affected by the baffle-plate air-hole diameter across different air–fuel ratios, with the thermal input maintained at 32 kW. At an air–fuel ratio of 15, the flame occupies approximately 38%, 32%, 30%, and 28% of the combustor length for air-hole diameters of 10, 12, 14, and 15 mm, respectively, demonstrating a clear reduction in flame length with increasing hole diameter. The visible flame length was determined by tracking the flame tip through nine uniformly distributed viewing ports along the combustor wall. This parameter is of practical importance, as it defines the minimum combustor length required to ensure complete flame containment under the investigated operating conditions.

This trend demonstrates that increasing the air-hole diameter results in a reduction in visible flame length. Enlarging the hole size reduces the incoming air velocity for the same fuel mass flow rate, which contributes to a shorter flame. When the air-hole diameter is kept constant at 15 mm, increasing the AFR results in a steady reduction in the visible flame length. For this configuration, the flame occupied approximately 28%, 24%, 20%, and 17% of the combustor length at different AFR values of 15, 20, 25, and 30, respectively. These results clearly demonstrate that higher AFRs, at an unchanged air-hole diameter, produce progressively shorter flames. The 10 mm air-hole configuration showed a comparatively narrow flammability window, sustaining stable operation only at AFR values of 15 and 20. The observed reduction in visible flame length with increasing air-hole diameter is primarily governed by the associated decrease in air exit velocity and jet momentum. Larger air holes generate slower and broader air jets, which weaken shear-layer development and reduce turbulent entrainment, thereby lowering near-field mixing intensity and completing combustion over a shorter axial distance^33,34^. The reduction in visible flame length with increasing air-hole diameter is attributed to enhanced early-stage air entrainment and mixing, which promotes faster fuel consumption and shortens the axial extent of the luminous reaction zone.

Analysis of the experimental results shows that flame length is mainly influenced by the diameter of the baffle-plate air passages and by the air–fuel ratio. Based on these findings, an empirical relation was developed to estimate flame length in terms of d_a_ and AFR. This correlation is valid for all test conditions examined in this work and is provided in the form shown in Eq. (3).

3 $$\:{\mathrm{L}}_{\mathrm{f}\left(\mathrm{E}\mathrm{q}\mathrm{u}.\right)}={\mathrm{a}}_{0}\mathrm{*}{\mathrm{d}}_{\mathrm{a}}^{{\mathrm{a}}_{1}}\mathrm{*}(\mathrm{A}\mathrm{F}{\mathrm{R})}^{{\mathrm{a}}_{2}}$$

The empirical correlation yielded coefficients a_o_ = 410.847, a_1_ = − 0.5799, and a_2_ = − 0.6637. To evaluate its performance, the model was applied to flame-length predictions for baffle-plate air-hole diameters of 10, 12, 14, and 15 mm across AFR values of 15, 20, 25, and 30, at 32 kW thermal load. The predicted results exhibited very good agreement with the experimental measurements, with an average Mean Absolute Percentage Error (MAPE) of approximately 2.5%. This low deviation confirms the reliability and applicability of the proposed correlation within the investigated operating conditions.

### The species concentrations

**Fig. 12:**
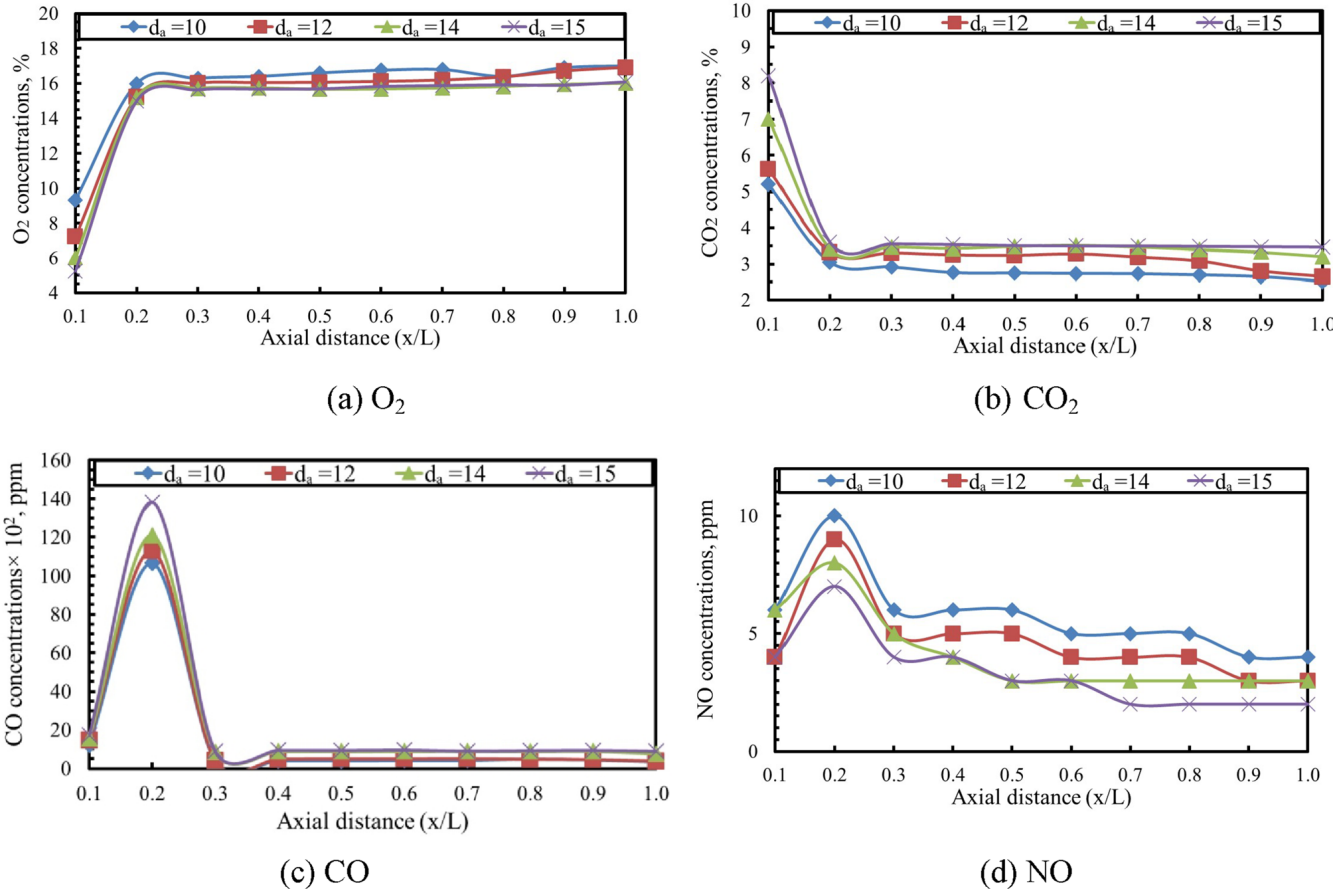
Effect of d_a_ on axial species concentrations [AFR = 15 and thermal load = 32 kW].

**Fig. 13:**
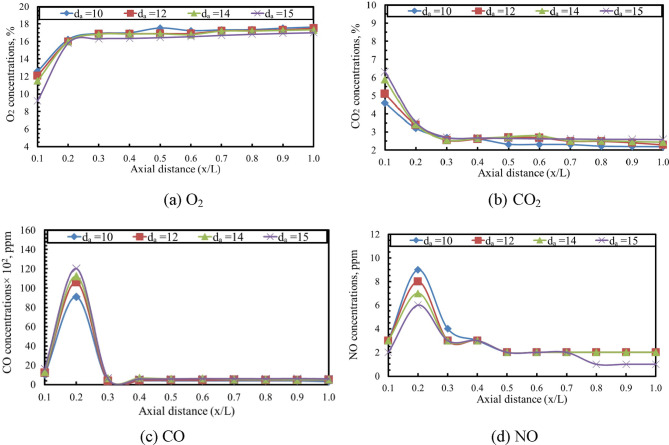
Effect of d_a_ on axial species concentrations [AFR = 20 and thermal load = 32 kW].

The axial concentrations of O₂, CO₂, CO, and NO in the exhaust gases were measured using a portable gas analyzer (Testo 350), which provides high-accuracy diagnostics for combustion studies. As shown in Figs. 12 and 13, the axial distributions of these species for different baffle-plate air-hole diameters are presented for air–fuel ratios of 15 and 20, respectively. Gas samples were extracted along the axial direction of the combustion chamber at uniform intervals of 50 mm through dedicated sampling ports integrated into the combustor. Prior to each measurement campaign, the gas analyzer was calibrated using zero and span calibration gases in accordance with the manufacturer’s recommendations. The extracted samples were conditioned to remove moisture before analysis to ensure measurement accuracy. Analyzer response time was accounted for, and all measurements were recorded after signal stabilization. At each axial location, the reported values represent time-averaged concentrations obtained over a fixed sampling duration to minimize fluctuations and enhance data reliability.

The results indicate that larger air-hole diameters correspond to lower axial O₂ concentrations. In contrast, the axial concentrations of CO₂ and CO increase along the combustion flame with larger air hole diameters. Moreover, the NO concentration was found to decrease with increasing air hole diameter, primarily due to the reduction in flame temperature associated with larger hole sizes. The findings also show that as the combustion process develops, the axial oxygen level drops in the vicinity of the flame front. Consequently, the oxygen level increases again toward the flame tip. On the other hand, the axial concentrations of CO₂, CO, and NO rise, attaining their maximum values near the flame front, where the axial temperature is highest, before gradually diminishing toward the end of the flame. This behavior closely follows the axial temperature profile and is consistent with observations reported in previous studies^47–49^. By comparing Figs. 12 and 13, it is evident that increasing AFR from 15 to 20 results in higher axial concentrations of O₂ and lower concentrations of CO₂, CO, and NO along the flame. This trend is primarily attributed to the decrease in flame temperature that occurs as the AFR increases. The CO concentration reaches its highest level at axial distance (x/L) = 0.2, after which it gradually decreases toward the end of the combustor. Moreover, the NO concentration attains its peak value at x/L = 0.2, followed by a gradual decline toward the downstream end of the combustor. This trend is observed for all baffle plate configurations. The observed reduction in NO concentration with increasing air-hole diameter is primarily a consequence of the lower peak flame temperatures, which suppress thermal NO formation. However, the decrease in CO concentration reflects enhanced oxidation in regions of improved mixing and longer residence time when smaller air hole diameter is used.

**Fig. 14:**
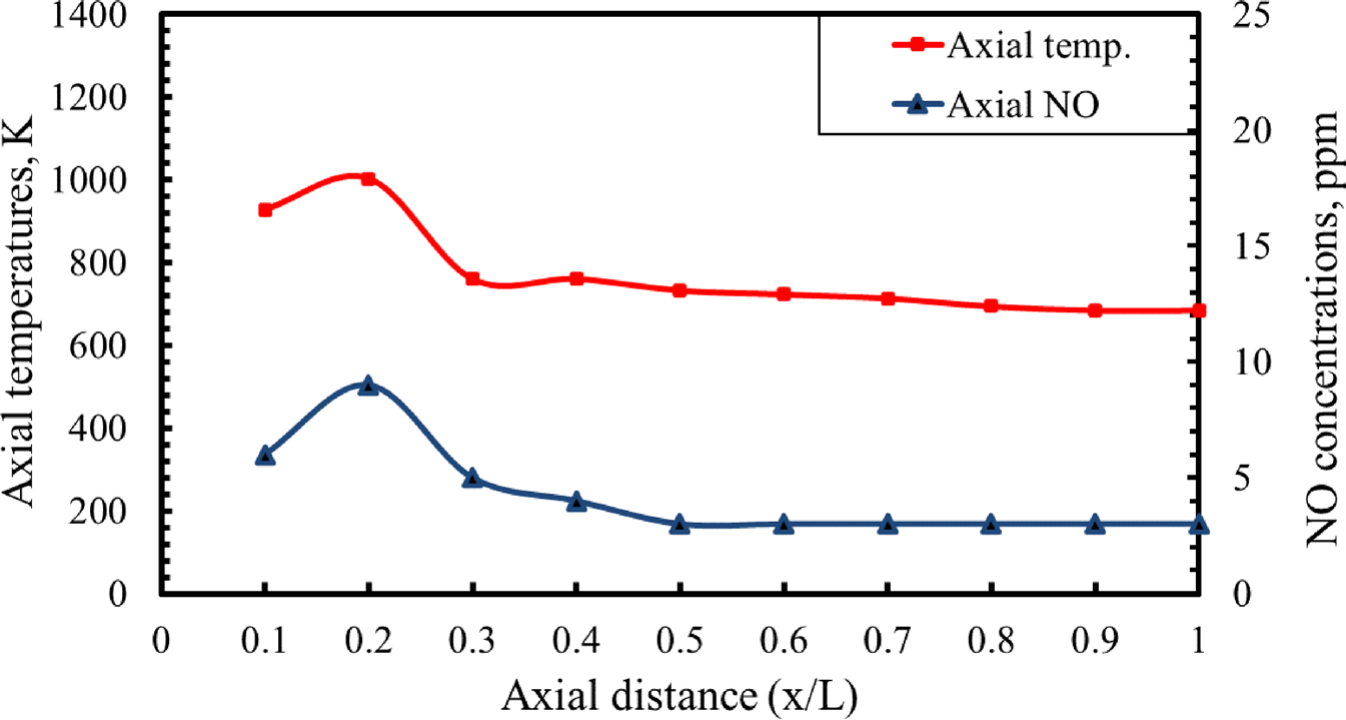
Axial distribution of NO concentration alongside axial flame temperature for an air hole diameter (dₐ) of 14 mm, AFR = 15.

According to Ibrahim et al.^28^, the NO concentration pattern closely mirrors the axial temperature profile of the flame. In particular, NO levels increase as the temperature rises, attaining their highest values near the flame front, exactly at the location of the maximum axial flame temperature, as illustrated in Fig. 14.

### Combustion efficiency

Combustion efficiency represents an important indicator for evaluating the overall effectiveness of a combustion system. It accounts for all measured variables and thermodynamic effects, including the flame-temperature field and the different modes of heat loss, such as energy carried away by the hot exhaust stream, absorbed by the coolant, or dissipated to the environment through heat transfer. The combustion efficiency ($\:{\eta\:}_{comb.}$) is expressed as the ratio between the total useful thermal energy released, covering contributions from exhaust-gas heat, radiative and convective losses, and the energy losses in the cooling water due to heat transferred and the total energy supplied to the system. The input energy which represents the energy made available for the combustion process is determined by multiplying the fuel heating value by the fuel mass-flow rate. Based on these considerations, the combustion efficiency can be written as:

To quantify the energy absorbed by the cooling water, the following relation was applied:

4 $$\:{\dot{\mathrm{Q}}}_{\mathrm{c}\mathrm{w}}={\dot{\mathrm{m}}}_{\mathrm{c}\mathrm{w}}\mathrm{*}\:{\mathrm{C}}_{\mathrm{P}\mathrm{w}}\:\:{(\mathrm{T}}_{\mathrm{w}\left(\mathrm{o}\mathrm{u}\mathrm{t}\right)}-{\mathrm{T}}_{\mathrm{w}\left(\mathrm{i}\mathrm{n}\right)})$$

In this relation, the temperature rises of the cooling water, from $\:{T}_{w\left(in\right)}$ at the inlet to $\:{T}_{w\left(out\right)}$ at the outlet, together with its mass flow rate $\:{\dot{m}}_{cw}$ and the specific heat of water $\:{C}_{Pw}$, is used to determine the heat absorbed by the coolant, denoted as $\:{\dot{\mathrm{Q}}}_{\mathrm{c}\mathrm{w}}$ (kW).

To quantify the energy carried away by the exhaust gases, the following equation was applied:

5 $$\:{\dot{\mathrm{Q}}}_{\mathrm{e}\mathrm{x}\mathrm{h}\mathrm{a}\mathrm{u}\mathrm{s}\mathrm{t}}={\dot{\mathrm{m}}}_{\mathrm{g}}\mathrm{*}\:{\mathrm{C}}_{\mathrm{P}\mathrm{g}}\:{\:(\mathrm{T}}_{\mathrm{g}}-{\mathrm{T}}_{\mathrm{a}})$$

where $\:{\dot{Q}}_{exhaust}$ represents the thermal energy transported by the exhaust gases (kW), $\:{\dot{m}}_{g}$ is the exhaust-gas mass flow rate (kg/s), $\:{T}_{a}$ denotes the ambient temperature (K), $\:{\:T}_{g}$ is the measured average temperature at the exhaust stream (K), and the $\:{C}_{Pg}$ denotes the exhaust gases specific heat represented in (J/kg·K),

Heat transfer by convection is calculated using the following equation:

6 $$\:{\dot{Q}}_{convection}=\:{A}_{s}\:{*h\:\:(T}_{wall}-{T}_{a})$$

Where $\:{A}_{s}$ is the combustor outer surface area (0.314 m^2^), ​ h is the convection heat transfer coefficient, ranging from 3 to 4.72 W/m^2^·K, determined as a function of the Nusselt number, T_wall_ is the measured combustor wall temperature in K, and T_a_ is the measured ambient temperature in K.

The calculation of radiative heat transfer is carried out by the following expression:

7 $$\:{\dot{Q}}_{radiation}=\:{A}_{s}*\sigma\:*\epsilon\:\:\:({T}_{wall}^{4}-{T}_{a}^{4})$$

Where $\:{A}_{s}$ is the combustor outer surface area (0.314 m^2^), ​ $\:\sigma\:$ is the Stefan-Boltzmann constant (5.67 × 10⁻⁸ W/m²K⁴), $\:\epsilon\:$ is the emissivity of the combustor wall (0.35).

The combustion efficiency was determined using the following equation:

8 $$\:{\eta\:}_{comb.}=\:\frac{{\dot{Q}}_{cw}+\:{\dot{Q}}_{exhaust}+{\dot{Q}}_{radiation}+{\dot{Q}}_{convection}}{\mathrm{H}\mathrm{e}\mathrm{a}\mathrm{t}\:\mathrm{i}\mathrm{n}\mathrm{p}\mathrm{u}\mathrm{t}}\times\:100$$

where $\:{\dot{Q}}_{radiation}$ is the radiation heat loss in kW, and $\:{\dot{Q}}_{convection}$ is the convection heat loss in kW.

The equations were applied together with the experimental measurements to quantify the thermal losses, and this information was subsequently integrated into the comprehensive evaluation of combustion efficiency, in accordance with the methodology outlined in^50–52^. To evaluate combustion efficiency, data from several key measurements were utilized, including the exhaust flue gases temperature, the temperatures of cooling-water at outlet and inlet, the cooling-water mass flow rate, and the combustor outer surface temperature. The wall-temperature readings, in particular, serve as an indicator of the heat dissipated to the surroundings through convective and radiative losses.

**Fig. 15:**
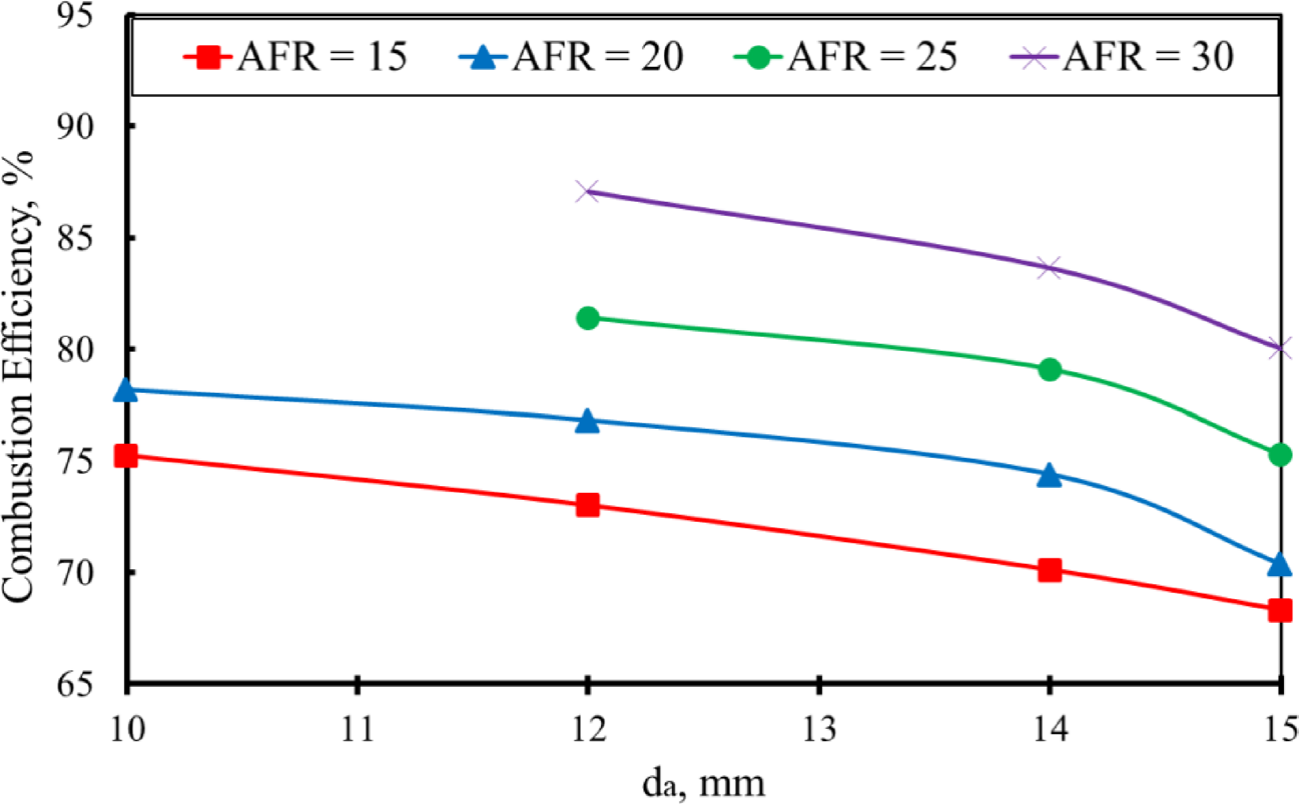
Effect of d_a_ on combustion efficiency across various AFRs [T.L. = 32 kW].

As shown in Fig. 15, the baffle-plate air-hole diameter (d_a_) has a pronounced influence on combustion efficiency across the investigated range of air–fuel ratios at a constant thermal input of 32 kW. The results indicate that increasing the air-hole diameter leads to a systematic reduction in combustion efficiency. Specifically, when d_a_ is increased from 10 mm to 15 mm, the combustion efficiency decreases by approximately 10.17% at AFR = 15 and 11.04% at AFR = 20. Although the configuration with d_a_= 10 mm exhibits a relatively narrow flammability range, limiting stable operation to AFR values of 15 and 20, it achieves higher combustion efficiency within this range. Furthermore, increasing d_a_ from 12 mm to 15 mm results in an efficiency reduction of about 8.1% at AFR = 25 and approximately 8.07% at AFR = 30. Overall, combustion efficiency is observed to improve with increasing air–fuel ratio for all tested baffle-plate configurations. The reduction in combustion efficiency at larger air-hole diameters results from increased heat losses associated with enhanced wall recirculation and exhaust-gas dilution, which reduce the fraction of released chemical energy converted into useful thermal output.

Although the present experiments were conducted at a fixed thermal input of 32 kW, the observed trends associated with air-hole diameter and air–fuel ratio are governed primarily by air-jet momentum, mixing characteristics, and recirculation characteristics.

By targeting previously overlooked aspects in numerical analyses, the present experimental investigation enhances current knowledge of combustion behavior. While earlier research predominantly relied on computational simulations and lacked empirical validation, especially concerning exhaust emissions, the present work introduces a comprehensive experimental framework that evaluates both combustion performance and exhaust gas emissions. In contrast to earlier work, this study provides a detailed examination of combustion-generated species, with special emphasis on the levels of CO and NO present in the exhaust stream. These emissions measurements offer detailed insights into combustion efficiency and the mechanisms of pollutant formation under various operating conditions.

A statistical comparison with previously published experimental data was carried out to evaluate the reliability of the present results. The maximum flame temperature and CO concentration measured in this study, 1147 K and 298 ppm, respectively, were compared with the values reported by Gad et al.^52^, who investigated LPG combustion operating conditions closely matching those of the present study, using a baffle plate with N_a_ = 4, R_a_ = 0.25D, and d_a_/d_n_ = 2.5. The resulting deviations were approximately 1.2% for the maximum flame temperature and 3.8% for CO concentration, which fall within acceptable experimental uncertainty limits and demonstrate good agreement with the literature.

The alignment of the current results with established trends reported in the literature reinforces the validity of the adopted experimental methodology. Furthermore, the inclusion of exhaust gas analysis and the broader range of performance indicators represents a significant advancement, supplying reliable benchmark data to support and refine future numerical models. A detailed comparison between this work and previous studies is provided in Table 2.

**Table 2:** Comparative overview of this work relative to past studies.

Reference	Type of Fuel	Study	*N*_a_	*R*_a_	Ф	NO, PPM	CO, PPM	L_f_/d_*n*_	η_comb_,	T_max_, K
									%	
Kim and Park^36^	H_2_	Numerically	8	0.25D	0.5	NA	NA	24	97	1883
Kim and Park^39^	CH_4_	Numerically	6	0.321D	0.7	NA	NA	24.4	95.8	1960
Kim and Park^35,37,38^	CH_4_	Numerically	6	0.286D	0.5	NA	NA	37.5	97.5	1450
					0.7			32	92	1720
Gad et al.^52^	LPG	Experimentally	4	0.25D	0.5	12	310	30	66.9	1162
Present study	LPG	Experimentally	8	0.25D	0.5	1	298	17.5	87	1147
					0.75	2	356	21.6	76.8	1202

## Conclusions

This study provides a detailed investigation into how variations in the baffle-plate air-hole diameter influence the behavior of the flame. A series of experiments was performed at constant thermal load of 32 kW using air-hole diameters of 8, 10, 12, 14, and 15 mm, combined with AFR settings of 15, 20, 25, and 30. The principal findings drawn from these experiments are outlined below:

- An increase in the air hole diameters of the baffle plate results in a wider flame stability region, indicating enhanced flame anchoring and reduced susceptibility to blow-off.
- The maximum flame temperature increases as d_a_ decreases at the same AFR. It was measured at AFR = 20 for d_a_ = 10 mm, reaching a value of about 1232 K, about 1.3%, 2% and 4.64% higher than d_a_ = 12, da = 14, and 15 mm, respectively.
- The visible flame length decreased as the air hole diameters increased at the same AFR. The flame lengths measured at AFR = 15 were about 38%, 32%, 30%, and 28% of the total combustor length at d_a_ = 10, 12, 14, and 15 mm, respectively.
- By increasing the AFR, the visible flame length decreased at the same d_a_. The flame lengths measured at d_a_ = 15 mm are 28%, 24%, 20%, and 17% of the combustor length for AFR values of 15, 20, 25, and 30, respectively.
- The axial concentrations of the carbon monoxide (CO) decrease along the combustion flame as the diameter of the air hole increases.
- The NO concentration decreases with increasing air hole diameter, which occurs as a result of the reduction in flame temperature caused by increased hole diameters.
- An empirical correlation for the flame length as a function of da and AFR was shown with an average error between the measured and calculated values of about 2.5%.
- The combustion efficiency decreased with increasing air hole diameters. Specifically, da increased from 10 mm to 15 mm, the combustion efficiency dropped by approximately 10.17% at AFR = 15 and 11.04% at AFR = 20.

## Data Availability

Data are available from the corresponding author upon reasonable request.

## Abbreviations

AFR: Air fuel mass ratio
${C}_{Pg}$: Specific heat capacity of the exhaust gases, J/kg·K
${C}_{Pw}$: Specific heat capacity of water, J/kg·K
D: Combustor inner diameter, mm
d_a_: Air hole diameter, mm
d_n_: Nozzle fuel diameter, mm
d_a_/d_n_: Dimensionless air hole diameter to fuel diameter
L: Combustor length, mm
L_f_: Flame length, mm
L_f_/ d_n_: Dimensionless flame length
LPG: Liquefied petroleum gas
N_a_: Number of air holes
R: Combustor inner radius, mm
R_a_: Radial air hole positions for baffle plate, mm
r/R: Dimensionless radial distance
$${T}_{a}$$: Ambient temperature, K
${T}_{w\left(in\right)}$: Inlet water temperature, °C
${T}_{w\left(out\right)}$: Outlet water temperature, °C
T.L: Input thermal load, kW
x/L: Dimensionless axial distance
${\dot{m}}_{cw}$: Mass flow rate of cooling water, kg/s
${\dot{m}}_{f}$: Fuel mass flow rate, g/s
${\dot{m}}_{g}$: Mass flow rate of exhaust gases, kg/s
${\dot{Q}}_{convection}$: Heat transferred by convection, kW
${\dot{\mathrm{Q}}}_{\mathrm{cw}}$: Heat transfer to the cooling water, kW
${\dot{Q}}_{exhaust}$: Heat carried by exhaust gases, kW
${\dot{Q}}_{radiation}$: Heat transferred by radiation, kW
$${\eta }_{comb}$$: Combustion efficiency
Ф: Equivalence ratio
